# Investigation of *Acinetobacter baumannii* Activity in Vascular Surgery Units through Epidemiological Management Based on the Analysis of Antimicrobial Resistance, Biofilm Formation and Genotyping

**DOI:** 10.3390/ijerph18041563

**Published:** 2021-02-07

**Authors:** Anna Szczypta, Katarzyna Talaga-Ćwiertnia, Małgorzata Kielar, Paweł Krzyściak, Anna Gajewska, Mirosław Szura, Małgorzata Bulanda, Agnieszka Chmielarczyk

**Affiliations:** 1 Faculty of Medicine and Health Sciences, Andrzej Frycz Modrzewski Krakow University, 30-705 Kraków, Poland; infoepid@interia.pl; 2 The Bonifratri Order Hospital of St. John Grande, 31-061 Kraków, Poland; miroslaw.szura@uj.edu.pl; 3 Jagiellonian University Medical College, Faculty of Medicine, Chair of Microbiology, Department of Infection Control and Mycology, 31-008 Kraków, Poland; pawel.krzysciak@uj.edu.pl (P.K.); malgorzata.bulanda@uj.edu.pl (M.B.); 4 Medical Diagnostic Laboratory with a Bacteriological Unit, St. Louis Regional Specialised Children’s Hospital, 31-503 Kraków, Poland; gkielar@o2.pl; 5 Oncogene Diagnostics, 31-546 Kraków, Poland; a.mroczek@poczta.onet.pl; 6 Jagiellonian University Medical College, Department of Clinical and Experimental Surgery, 31-008 Kraków, Poland; 7 Jagiellonian University Medical College, Faculty of Medicine, Chair of Microbiology, Department of Bacteriology, Microbial Ecology and Parasitology, 31-008 Kraków, Poland; agnieszka.chmielarczyk@uj.edu.pl

**Keywords:** *Acinetobacter baumannii*, extensive drug resistance, ST2 clone, OXA-23, epidemiological investigation, hospital outbreak

## Abstract

Background/Objectives: The genus *Acinetobacter* demonstrates resistance to antibiotics and has been shown to spread in the hospital environment causing epidemic outbreaks among hospitalized patients. The objectives of the present study was to investigate the antibiotic resistance, biofilm formation, and clonality among *Acinetobacter baumannii* strains. Materials and Methods: The study involved 6 (I Outbreak) and 3 (II Outbreak) *A. baumannii* strains isolated from patients hospitalized in vascular surgery unit. Results: All tested *A. baumannii* strains were extensively drug resistant (XDR) and all the isolates were carbapenem-resistant and among them, all carried the *bla*_OXA-51_ gene, the *bla*_OXA-24_ gene, as well as the *bla*_OXA-23_ gene. All of the investigated strains had the ability to form a biofilm, but all of them produced less biofilm than the reference strain. Multi-locus sequence typing (MLST) showed that all strains belonged to the ST2 clone. Pulsed-field gel electrophoresis (PFGE) divided the tested outbreak strains into two clones (A and B). Conclusion: This study shows a nosocomial spread of XDR *A. baumannii* ST2 having the *bla*_OXA-51_ gene, the *bla*_OXA-24_ gene, as well as the *bla*_OXA-23_ gene, low biofilm formers, that was prevalent in the vascular surgery unit. To identify the current situation of vascular surgery departments targeted epidemiological investigation was needed. Effective implementation of infection control prevented the spread of the epidemic outbreaks.

## 1. Introduction

*Acinetobacter baumannii* has become one of the most difficult healthcare associated infection pathogens to control and treat. It has, recently, demonstrated a rapid increase in resistance to antimicrobials, being multidrug-resistant (MDR) and extensively drug-resistant (XDR) [1]. *A. baumannii* can cause pneumonia, bloodstream, urinary tract, and surgical site infections [2,3]. It has been shown to spread in the hospital environment causing epidemic outbreaks among hospitalized patients because of its ability to colonize the skin as well as medical devices and the respiratory tract of patients and health care workers [4,5]. It also has the ability to form biofilm on inanimate surfaces [2,6]. The potential to form biofilm and the possession of a drug resistance mechanism seem to be the way to enhance viability in the hospital environment [7]. 

The resistance of *A. baumannii* strains in Poland has been constantly on the rise for many years. Currently, according to the data from the European Antimicrobial Resistance Surveillance Network (EARS-Net), the Polish isolates of *Acinetobacter* sp. resistant to carbapenems, fluoroquinolones and aminoglycosides constitute 62.9%, which puts our country among several European countries with a high percentage of resistant strains (Latvia 56.8%, Bulgaria 66.4%, Italy 75.7%, Romania 77.6%, Cyprus 78.2%, Greece 81.3%, Lithuania 85.1%, Croatia 90.8%) [8]. Pandrug resistant bacteria (PDR) and XDR isolates of *A. baumannii* have been reported for many years, not only in Polish hospitals, but also globally [9,10,11,12,13]. Infections caused by these strains are a serious therapeutic issue and are particularly common among intensive care unit (ICU) patients, but also appear in people hospitalized in other departments [14,15,16,17,18,19]. It seems that *A. baumannii* is one of the main etiologic agents of hospital associated infections (HAIs) and epidemic outbreaks in Poland, compared to infections caused by other non-fermenting bacteria (*Pseudomonas aeruginosa*), which are less frequently reported in our country [9,20,21]. For two decades in Poland, *A. baumannii* infections have been a significant proportion of healthcare-associated infections (especially in ICUs) but there are only a few reports from Poland characterizing these pathogens and the conditions of epidemic outbreaks [20,22,23]. 

The main purpose of this study was to characterize the *A. baumannii* strains causing hospital outbreaks in one of the hospitals in Kraków that occurred in 2017 and 2018. The second aim was to evaluate the management of the *A. baumannii* outbreaks.

## 2. Materials and Methods

### 2.1. Bacterial Isolates

The study involved 6 (I Outbreak, IO) and 3 (II Outbreak, IIO) clinically significant non-duplicate *A. baumannii* isolates isolated from surgical site infections (SSIs) and wounds derived from patients hospitalized in the vascular surgery unit in a specialist hospital in Kraków. Species identification was carried out with biochemical tests (Neferm Test Erba Lachema Polska) and then verified by a matrix-assisted laser desorption ionization time-of-flight mass spectrometry (MALDI-TOF) biotyper (Bruker Daltonik GmbH, Bremen, Germany).

### 2.2. Antibiotic Resistance

Antimicrobial sensitivity testing was performed using the disk diffusion method. The following antimicrobials were tested (all discs were from Oxoid): ceftazidime (CAZ 10 mg), imipenem (IMP 10 mg), meropenem (MEM 10 mg), ciprofloxacin (CIP 5 mg), levofloxacin (LEV 5 mg), amikacin (AK 30 mg), gentamicin (GN 10 mg), tobramycin (TB 10 mg), netilmicin (NT 10 mg), and trimethoprim–sulfamethoxazole (SXT 1.25/23.75 mg), excepting evaluation of the minimum inhibitory concentrations (MICs) of colistin by the broth microdilution (MIC-Strip Colistin, Merlin). Drug susceptibility was interpreted according to the guidelines of the European Committee on Antimicrobial Susceptibility Testing (EUCAST) guidance (clinical breakpoint tables v.8.0; www.eucast.org/clinical_break points/ (accessed on 6 February 2021)) [24]. XDR strains were defined as those strains that were susceptible to no more than two antimicrobial classes [25].

### 2.3. Screening for Metallo-β-Lactamase Phenotype

The metallo-β-lactamase (MBL) mechanism of resistance was detected by double-disk synergy test (DDST) with an EDTA disk (10 µL 0.5 M EDTA, pH 7.3–7.5), disks of ceftazidime (CAZ 30 mg; Oxoid) and imipenem (IMP 10 mg; Oxoid) placed 20 mm apart from a disc containing EDTA. The test was considered positive if the inhibition zone around the disc with CAZ and/or IMP was enhanced [26]. 

### 2.4. Screening for Carbapenemase Type Beta-Lactamases Phenotype

The *Klebsiella pneumoniae* carbapenemases (KPC) phenotype of resistance was detected by combined disc test (CDT) with meropenem disks (MEM 10 mg; Oxoid) and boric acid (20 µL 15 mg/mL boric acid). The test was considered positive if the zones around the disc with MEM and MEM+boric acid differed by 7 mm (or more) [27].

### 2.5. Polymerase Chain Reaction 

DNA templates were extracted using a Genomic Mini kit (A&A Biotechnology, Gdynia, Poland) according to the manufacturer’s instructions. All isolates were screened for the presence of four MBL genes in a multiplex polymerase chain reaction (PCR): *bla*_VIM_ (390 bp), *bla*_IMP_ (188 bp), *bla*_SPM-1_ (271 bp), and *bla*_GIM-1_ (477 bp). PCR analysis was performed using previously published primers and conditions [28].

PCR was also used to screen for the four *bla*_OXA_ genes: *bla*_OXA-23_ (116 bp), *bla*_OXA-24_ (151 bp), *bla*_OXA-51_ (112 bp), and *bla*_OXA-58_ (141 bp) in *A. baumannii* isolates. To detect specific DNA sequences, the pairs of specific primers described by Huang et al. were used [29]. The insertion element IS*Aba*I (301 bp) located upstream of the *bla*_OXA-51_ gene (112 bp) was detected separately in a PCR reaction using primers and conditions as described by Pasanen et al. [30].

### 2.6. Biofilm Formation

A biofilm formation assay was carried out as described by Merritt et al. [31]. Biofilm formation was tested on the M63 medium (Amresco, Solon, OH, USA) supplemented with casein (Oxoid Ltd., Hampshire, UK) after 28 h. The obtained crystal violet absorbance values corresponded to the amount of biofilm produced by each strain. The mean absorbance values for the control wells for each microtiter plate were subtracted from the mean absorbance values for each strain, allowing all strains to be compared on parameters of variable distribution (mean and SD) with the biofilm-forming *A. baumannii* ATCC 19606 reference strain.

### 2.7. Multi-Locus Sequence Typing

Multi-locus sequence typing (MLST) was performed as described by Diancourt et al. [32] with a Pasteur scheme. Sequencing of housekeeping genes *cpn*60, *fus*A, *glt*A, *pyr*G, *rec*A, *rpl*B, *rpo*B was performed by Genomed SA (Warsaw, Poland), and the resulting sequences were analyzed using the ChromasPro 1.4 software (Technelysium Pty Ltd., South Brisbane, QLD, Australia). Sequence types (STs) were determined by comparison to the PubMLST database [33]. 

### 2.8. Pulsed-Field Gel Electrophoresis

Analysis of genetic similarity between *A. baumannii* strains was performed using pulsed-field gel electrophoresis (PFGE) in accordance with a previously published protocol [34]. Electrophoresis was conducted using the CHEF III PFGE system (Bio-Rad Laboratories, Inc., Hercules, CA, USA). Gel Compar II 6.5 (Applied Maths, Sint-Martens-Latem, Belgium) was used for cluster analysis using the Dice coefficient and unweighted pair group method with arithmetic mean. Isolates with more than 95% similarity were clustered together as identical.

### 2.9. Characteristics of Hospital

The vascular surgery department (VSD) occupies one floor in a historic hospital building established over 100 years ago. In 2017 and 2018, totals of 1832 and 1756 patients were admitted to the hospital, respectively. The hospital offers vascular surgery procedures such as non-bifurcated aortic prosthesis, aortic-bifemoral prosthesis, suprapubic extra-anatomical femoral prosthesis, and femoropopliteal prosthesis surgery, endarterectomy, percutaneous angioplasty with stent implantation, endovascular aortic repair, percutaneous transluminal angioplasty, thrombectomy, thromboendarterectomy, embolectomy. In 2017, as well as in 2018, there was a total of 44 beds in the VSD. There were 7 physicians present in the unit per working day and 1 on each night shift, and 11 nurses on each working day and 4 at night shifts. 

### 2.10. Epidemiological Investigation

To control and eliminate IO and IIO, epidemiological investigation and activities were undertaken by the infection control team (ICT), including the activity of an epidemiological nurse. In the epidemiological investigation, 34 swabs from the inanimate environment of the VSD (washbasin taps, soap dispensers, hand care lotion, dressing trolley, cleaning equipment, shower tray, soap, computer keyboard, blood pressure monitors, bed mattress, ECG machine, mop after washing, the air in the OR, surgical scrub sinks) and 11 tests from the healthcare workers (hand swabs from physicians and nurses) were taken. The samples were collected from surfaces of 10 cm^2^ area using sterile flocked swabs (Copan Liquid Amies Elution Swab; Copan Diagnostic Inc., Murrieta, CA, USA). Then the swabs were cultured as described by Różańska et al. [35]. Tests taken from healthcare workers hands were made on TSA Contact with Disinhibitor plus TWI plates (Thermo Fisher Scientific Inc., Waltam, MA, USA). The cultured species were identified with biochemical tests (Neferm Test; Erba Lachema s.r.o., Brno, Czech Republic) and then verified by MALDI-TOF biotyper (Bruker Nano GmbH; Berlin, Germany).

Healthcare workers’ hand hygiene and the use of personal protective equipment were evaluated according to the WHO guidelines on hand hygiene in health care [36]. Disinfection in the unit, including equipment disinfection, was monitored by an epidemiological nurse. The air in operating theaters was tested using the sedimentation method as described by Kaiser and Wolski [37] using TSA + neutralized/irradiated 90 mm plates (bioMérieux, Craponne, France). The plates were placed in three different heights, and the time of exposition was 30 min.

### 2.11. Statistical Methods 

Statistical analysis was performed using R Language and Environment for Statistical Computing software [38]. The significance level for all statistical tests was set at *p* ≤ 0.05. ANOVA analysis and the Tukey post hoc analysis were performed to compare every strain with each other (Tukey multiple comparisons of means). And also the Dunnett post hoc analyses were performed to compare each strain with the control strain.

All collected data entered into the database and analyzed during this study were previously anonymized and de-identified.

## 3. Results

### 3.1. Characteristics of Patients

Analysis of the patients population data showed that the median patient age in IO was from 64 to 82 year (mean: 73.5) and in the IIO was 59 to 81 (mean: 70). The majority of patients were female in IO (5 vs. 1), and male in IIO (2 vs. 1) (details in Table 1). All patients were hospitalized in the vascular surgery unit and operated on due to vascular disorders. The average length of stay was 7 days. All patients were treated with antibiotics before being operated, but we were unable to access this information. All patients in IO and all from IIO had surgical site infection (SSI). The patients 252/17 and 342/17 had SSI caused by HAI pathogens and were then colonized by an XDR *A. baumannii* strain after a few days. Patients from IO were cohorted in the same rooms in 2017 and those from IIO in one room in 2018. All patients were discharged from the hospital in good condition after both IO and IIO. 

### 3.2. Characteristics of A. baumannii Strains

All *A. baumannii* strains (n = 9, 100%) were resistant to all of the antimicrobials tested, with the exception of colistin, so that all were classified as XDR strains (details in Table 2). Susceptibility to colistin was shown by 8 (90%) *A. baumannii* strains, only one (no. 279/17; 10%) was resistant based on EUCAST recommendations. Neither the EDTA test nor the KPC screening test gave positive results. The presence of the *bla*_OXA-51_ gene, the *bla*_OXA-24_ gene, as well as the *bla*_OXA-23_ gene was confirmed in all *A. baumannii* strains tested. The *bla*_OXA-58_ gene, the IS*Aba*1 element or the *bla*_VIM-1_, *bla*_IMP_, *bla*_SPM-1_, and *bla*_GIM-1_ genes were not detected in any strain (details in Table 2). 

**Figure 1:**
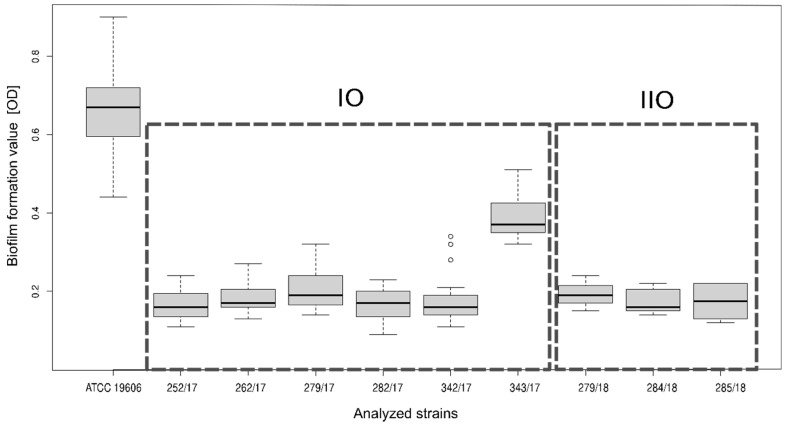
Comparison of biofilm formation by *A. baumannii* strains originating from IO and IIO. In the boxplots chart: the black line is the median, box represent interquartile range and the dots are outliers. The frames contain strains qualified for the first (IO) and second (IIO) outbreaks.

All (100%) of the investigated strains had the ability to form a biofilm, but all of them produced 3.5-fold less biofilm than the reference strain (post hoc Dunnett analysis) (details in Figure 1). Comparison of biofilm production by strains isolated from IO and IIO showed that there was no difference in biofilm production, except strain 343/2017, which produced more biofilm than other strains (post hoc Tukey analysis) but still less than the reference strain (details in Figure 1). 

**Figure 2:**
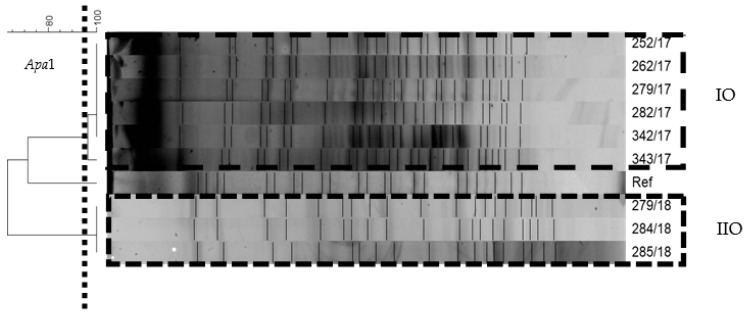
Pulsed-field gel electrophoresis dendrogram of *A. baumannii* strains belonging to IO and IIO, generated by the Gel Compar II software (Dice coefficient, tolerance 1%, optimization 1%). The scale indicates a percent similarity of the pulsed-field gel electrophoresis (PFGE) profiles. The dotted line indicates 95% similarity values of the PFGE profiles. The frames contain strains qualified for the first (IO) and second (IIO) outbreaks. Ref is the *A. baumannii* ATCC 19606 reference strain, *Apa*1 is the cutting enzyme.

The MLST method revealed that all 9 *A. baumannii* strains belonged to ST2 according to the Pasteur scheme. The PFGE method indicates that the five isolates within the IO had the same pulsotypes, and the strain 343/17 was 96% similar to these strains (A clone). In the IIO, there was 100% similarity between the tested strains (B clone). The strains from IO and IIO were more diverse. The IO and IIO were induced by two different strain clones (Figure 2). 

### 3.3. Epidemiological Investigation

**Figure 3:**
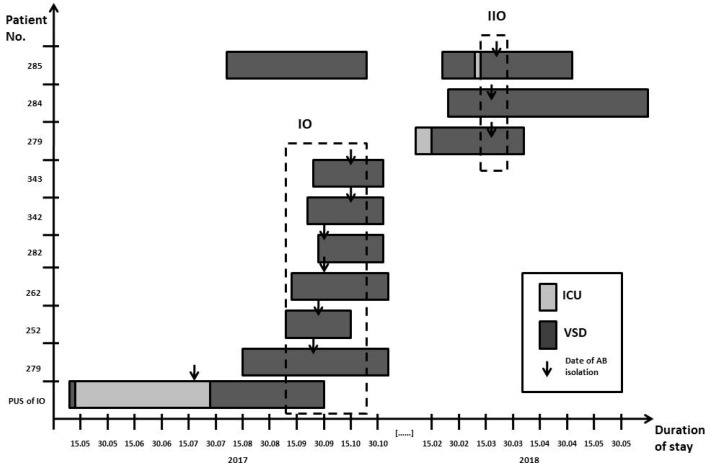
Outbreak measures in the timeline of the two outbreaks causing by XDR *A. baumannii* isolates. ICU—intensive care unit, VSD—vascular surgery department, IO—first outbreak, IIO—second outbreak, AB—*A. baumannii*. The arrow marks the date of isolation of *A. baumannii* strains. The frames represent the duration of the first (IO) and second (IIO) outbreaks.

The epidemiological investigation found a possible unconfirmed source patient (PUS), who was hospitalized in the ICU in July 2017 by ICT (Table 1, Figure 3). This patient zero had bronchial tree colonization with XDR *A. baumannii* (patient without symptoms of infection). The PUS patient was then transferred to the VSD and stayed there until the end of September 2017. All patients with SSI caused by XDR *A. baumannii* were hospitalized with the PUS patient in the VSD at the same time. Unfortunately, the PUS patient was not included in the epidemic outbreak because there was neither colonization with XDR *A. baumannii* nor another infection when she was transferred to the vascular surgery unit. Patients included in IO were placed in different rooms but they were looked after by the same staff in the ward. In September 2017, the bacteriology laboratory was notified of the first outbreak of *A. baumannii* in the VSD through an alert to the ICT. The first epidemic outbreak lasted from 11 September 2017 to 20 October2017. To eliminate the first outbreak, many activities were undertaken. During the IO, patients infected and colonized with XDR *A. baumannii* were cohorted separately in rooms with bathroom facilities. Dedicated physicians and nurses were chosen to look after patients qualified for IO. For 1 month, admissions to the surgical ward were halted. In the epidemiological investigation, 34 hospital inanimate environment examinations (washbasin taps, soap dispensers, hand care lotion, dressing trolley, cleaning equipment, shower tray, soap, computer keyboard, blood pressure monitors, bed mattress, ECG machine, mop after washing, the air in the OR, surgical scrub sinks) and 11 tests from the healthcare workers (hand swabs from physicians and nurses) were performed. The air in the operating theaters was tested by the sedimentation method and the result was negative. 

No *A. baumannii* strain was isolated from any of the swabs taken from the inanimate environment (operating block, VSD) or the hands of healthcare workers. In five cases, pathogenic bacteria (*Escherichia coli*, *Chryseobacterium* sp.) were found on the hands of medical workers. *Acinetobacter haemolyticus* was isolated from the hands of one physician. Methicillin-resistant *Staphylococcus aureus* (MRSA) and *Stenotrophomonas* sp. were isolated from the surfaces of various equipment. Moreover, *A. haemolyticus* was isolated from the service trolley, *Alcaligenes faecalis* from the shower tray, and MRSA was isolated from the cream dispenser. The soap and other equipment tested were sterile. 

In IIO, two patients presenting infection with *A. baumannii* were detected on 13 March 2018 (279/18, 284/18) and the third on 17 March 2018 (285/18). The patient 285/2018 was also hospitalized earlier in 2017 during the IO outbreak (Table 1, Figure 3). It was not possible to suspend patient admissions, so patients with SSI caused by *A. baumannii* were cohorted in one patient room, but dedicated physicians and nurses were chosen to look after patients qualified for IIO.

In the epidemiological investigation during the second outbreak, hospital inanimate environment examinations (the same as in the IO) and tests from the healthcare workers were performed. No *A. baumannii* strain was isolated from any of the swabs taken from the inanimate environment or the hands of healthcare workers. Only pathogenic bacteria as *E. coli* and *MRSA* were found on the hands of two medical workers. Moreover, *Enterococcus faecalis* from the shower tray was isolated. Other equipment tested were sterile. The air in the operating theaters was also tested, but the result was negative.

Since 2018, no outbreak caused by *A. baumannii* has occurred in the VSD or any other hospital units.

### 3.4. Outbreak Response

In response to the first outbreak, compliance with the sanitary and epidemiological regime in the unit was tested and recommendations on the conduct in the ongoing outbreak were issued in writing by the ICT (data not shown). Secondly, healthcare workers from the vascular surgery unit and the cleaning company were trained on the sanitary and hygienic regime. Then, isolation procedures were inspected several times during the epidemic.

During the IO, decontamination of the unit’s environment was performed using preparations containing hydrogen peroxide, chlorides, and quaternary ammonium compounds for large surfaces; preparations containing chlorides, and cyanuric acid or sodium bisulfate and sodium tetraborate were used for sinks and toilets and an alcohol-based preparation was applied for rapid disinfection of small surfaces. During the outbreak, fumigation with vaporized hydrogen peroxide hydrogen (VHP) was carried out in operating rooms and the unit, thorough cleaning and disinfection of all rooms and equipment in the vascular surgery unit were also performed. The epidemiological nurse monitored hand hygiene, the use of personal protective equipment, and disinfection in the unit, including equipment disinfection. Sustained implementation of the recommended infection prevention practices was observed. In October, the unit experienced cessation of transmission and the last efforts to extinguish the outbreak took place on 31 October.

In response to the IIO, compliance with the sanitary and epidemiological regime in the unit was tested, and recommendations on the ongoing outbreak were issued. Healthcare workers from the VSD and the cleaning company were trained on the sanitary and hygienic regime. During the IIO outbreak, decontamination of the unit’s environment and equipment in the vascular surgery department was performed. The cohortative room was fumigated with VHP after patients were discharged. The epidemiological nurse monitored hand hygiene, personal protective equipment, and disinfection in the unit.

## 4. Discussion

The epidemic outbreaks analyzed in our study were caused by the strains of *A. baumannii* belonging to type ST2, which is a characteristic and dominant clone in Poland and Europe [39,40,41]. The ST2 clone was already isolated from previous epidemic outbreaks in hospitals of Małopolska and Silesia [9,23,42]. The clone belongs to international clone II (IC2), which is also characterized by the presence of carbapenem-hydrolyzing class D oxacillinases (OXA), which is the major mechanism of carbapenem resistance among *A. baumannii* and the spread of acquired *bla*_OXA_ genes is well known [43,44,45]. Carbapenem-resistant *A. baumannii* strains harboring the *bla*_OXA-58_ gene were the predominant type, among others, in Italy, Greece and Turkey from 1999 to 2009. Then, a huge change in harboring *bla*_OXA_ genes and the domination of the *bla*_OXA-23_ gene in *A. baumannii* strains was observed globally [46]. In our study, *A. baumannii* isolates accumulated three *bla*_OXA_ genes. All of them possessed the *bla*_OXA-51_ gene, as the naturally occurring OXA enzyme [47], which was detected previously among others by Chmielarczyk et al. [23]. The tested strains also harbored the *bla*_OXA-23_ gene as well as the *bla*_OXA-24_ gene, which was also characteristic for 19.2% of the strains in the study by Chmielarczyk et al. We had not detected the *bla*_OXA-58_ gene in any strain. In other studies in Poland, similarly, the *bla*_OXA-58_ gene was not detected [23,48]. The IS*Aba*1 gene, which also contributes to carbapenem resistance, was not detected among any our study strains. It seems to be rare in Polish *A. baumannii* strains, as it was also detected previously in 10 strains out of the total of 125 tested [23]. Less frequently, carbapenem resistance is also mediated by MBLs, for example, VIM and IMP. In our study, neither the *bla*_VIM_, *bla*_IMP_, *bla*_SPM-1_, nor the *bla*_GIM-1_ genes were found. In a 2016 study by Chmielarczyk et al. [23], only two isolates possessed *bla*_VIM_ genes. Analysis of the presence of carbapenem resistance genes and a phenotypic assessment of susceptibility to all classes of antibiotics demonstrated that the strains isolated from both IO and IIO are extensively drug-resistant (XDR), which is another property characteristic of epidemic clone II. 

Responsibility for HAI is a significant health problem due to the limited options for antibiotic treatment of *A. baumannii* infections. Nowadays, in many cases, colistin is the key therapeutic option for *A. baumannii* infection treatment. It could be used alone or with other antimicrobials such as tigecycline, ampicillin-sulbactam, or carbapenems, if they are active [49]. In our study, all *A. baumannii* strains were XDR, with susceptibility to colistin (only 279/17 was resistant), so this therapeutic option was applicable. However, it is well-known that colistin therapy leads to the emergence of resistance to these antimicrobials. Furthermore, colistin therapy often causes side effects in patients [49].

Drug-resistant strains of *Acinetobacter* have been researched many times as flora that is persistent in hospitals around the world [50,51]. Drug resistance is a distinctive feature of HAI strains that linger in the hospital environment in which bacteria are under antibiotic pressure. It is possible for the *Acinetobacter* strains to survive in the hospital environment due to their ability to acquire and accumulate resistance to antibiotics employed in treatment, but also owing to the ability to produce biofilm [7,52]. In this study, the strains produced biofilm, however, it was very weak compared to the reference strain. Kaliterna et al. found, even more, that resistant clinical isolates did not form a biofilm [53]. In other previously done studies, researchers found that a large number of strong biofilm producers were drug-susceptible *A. baumannii* strains [7,54]. In our study, we found that one of the tested strains produced biofilm biomass stronger than others. It could be due to the fact that this strain differed slightly from the others in IO, which was also confirmed in the PFGE analysis.

In our opinion, epidemiological studies are important for monitoring the spread of XDR *A. baumannii* isolates in hospitals and should always be undertaken when an epidemic outbreak is detected. It is known that *Acinetobacter* may linger in the hospital environment and potentially be the source of HAI and hospital outbreaks, among others, due to their transient colonization of the hands of medical staff. In our study, what was detected on the hands of the staff was the species *A. haemolyticus* and other microbes representing the hospital microbiota, however, we did not detect the species *A. baumannii*. This may be indicative of only transient contamination of the hands of the staff or personal protective equipment. In other studies, the authors describe it as the way the epidemic spreads in healthcare centers [11,55,56]. In the study by Morgan et al. [57], the authors have also confirmed that it is not only the contamination of the hands of medical personnel, but also of gloves and coats, that is a potential route of transmission of nosocomial pathogens, especially the species *A. baumannii*. It was impossible for us to detect the source of *A. baumannii* isolates in the inanimate environment of the ward and the operating theater, which may suggest that the strains responsible for outbreaks came from patients (PUS patient). 

After analysis of the data available, PUS patient was most probably the source of the IO. It appears that the transmission of *Acinetobacter* strains and the development of epidemic outbreaks in hospitals may also result from transferring patients between departments [12,58]. In the situation analyzed by us, the patient rotation in the department of vascular surgery and hospitalization of patients in different units, e.g., transferring patients from the ICU to the department of vascular surgery (PUS patient), resulted in contact between patients qualified for IO and IIO. During the epidemic, the patients were staying in the same rooms, so it was possible to become colonized with the epidemic clone quickly. In the PFGE analysis, it was found that each outbreak was caused by independent clones (clone A and B in IO and IIO, respectively) which backs the argument that patient rotation in the unit is conducive to the development of epidemic outbreaks. 

It is also generally accepted that *A. baumannii* clones do not demonstrate such great and rapid variability as other microorganisms causing hospital epidemics (MRSA, VRE) [12,59,60]. Epidemic outbreaks caused by the same epidemic clone of *A. baumannii*, which occurred in long intervals (of over several months), had been described before [12,61]. 

Based on the molecular methods used, we conclude that the outbreaks and their sources were separate. The actions taken by the ICT after both, the first and the second outbreak were correct, effective, and produced the expected results.

In fact, the IO and IIO outbreaks triggered verification and improvement of hospital procedures and increased the personnel’s knowledge and awareness concerning the procedures limiting the risk of infection with *A. baumannii*. Decontamination of the vascular surgery unit and operating theatre environment using VHP was effective. In our opinion, the use of VHP was crucial in the attainment of infection control goals during IO and IIO. The importance of using the VHP in eradicating microorganisms of high environmental impact such as *A. baumannii* was emphasized in previous studies [61,62,63,64]. In our opinion, it was equally important that the hand hygiene policy education was provided not only for HCWs but also for the cleaning staff according to WHO guidelines [36]. The need for education on the proper hand hygiene procedure and its appropriate application is considered one of the most essential practices in limiting the number of epidemic outbreaks and preventing the spread of microorganisms in the healthcare environment [65,66,67]. What is more, Wang et al. [11] proved that foreign nursing workers, who were not employed by the hospital, or relatives of patients who care for them during their hospital stay may transmit bacteria between patients and may play an important role in the occurrence of outbreaks. In this context, we are strongly sure that the strategy to control the spread of resistant microorganisms, such as XDR isolates of *A. baumannii*, among patients, as well as the implementation of appropriate infection control measures and a surveillance program should prevent HAI infections and epidemic outbreaks in the future.

Our study has some limitations. For example, we had no data about previous outbreaks (i.e., outbreak from 2015) which could be important in more effective surveillance in this hospital, and for epidemiological studies in our region and country. We also had no XDR *A. baumannii* strain from PUS patient because it had not been frozen, so we could not conduct a complete epidemiological analysis of IO and IIO. Furthermore, we did not obtain data concerning antibiotic therapy, so it was impossible to determine the susceptibility of patients to infections and epidemic outbreaks caused by XDR *A. baumannii*.

In summary, this study shows a nosocomial spread of XDR *A. baumannii* ST2 having the *bla*_OXA-51_ gene, the *bla*_OXA-24_ gene, as well as the *bla*_OXA-23_ gene, low biofilm formers, that was prevalent in the vascular surgery department. To identify the current situation of the vascular surgery unit, targeted epidemiological investigation was needed. Due to phenotypic and molecular characterization of *A. baumannii* strains, controlling *A. baumannii* infections in hospitals presents a serious challenge. It should also be highlighted that the implementation of infection control effectively prevented the spread of epidemic outbreaks. It also proved that targeted investigation of molecular epidemiology and healthcare worker attitudes toward the outbreak of *A. baumannii* is important and implementation of infection control can effectively prevent the spread of nosocomial outbreaks caused by XDR *A. baumannii*.

## Figures and Tables

**Table 1:** Characteristics of patients included in the outbreak IO and IIO.

Patient No.	Age (Years)	Median (Mean) of Age (Years)	Sex	Duration of Hospital Stay	Ward (Room)	Infection Type	Accompanying/Preceding Disease	Outbreak No.
PUS of IO	72	-	F	11 May–12 May 2017 12 May–28 July 2017 28 July–29 September 2017	VSD ICU VSD	BTC	VAP, UTI, CRBSI (none of them due to *A. baumannii*), previous surgeries	unclassified
279	82	73.5 (73.5)	F	15 August–06 November 2017	VSD (217)	SSI	hypertension, COPD, CKD, DM, PAD, chronic ulceration	I
252	64		F	11 September–20 October 2017	VSD (218)	SSI	hypertension, DM, chronic ulceration	
262	81		F	15 September–03 November 2017	VSD (218)	SSI	hypertension, heart failure, ulceration	
282	66		M	29 September–30 October 2017	VSD (216)	SSI	hypertension, PAD, DM, previous surgeries	
342	66		F	19 September–30 October 2017	VSD (216,218)	SSI	PAD, ulceration, previous hospitalization in another hospital, DM2	
343	82		F	24 September–30 October 2017	VSD (204,207)	SSI	hypertension, PAD, CKD, DM, previous hospitalization and surgeries	
279	82	70.0 (70.3)	M	10 February–16 February 2018 16 February–13 April 2018	ICU VSD (216)	SSI	hypertension, antibiotic therapy due to SSI, DM2, previous hospitalization and surgeries	II
284	59		M	27 February–23 August 2018	VSD (209)	SSI	PAD, antibiotic therapy due to SSI, previous hospitalization	
285	70		F	9 August–24 October 2017 23 February–6 March 2018 6 March–9 March 2018 9 March–9 May 2018	VSD VSD (217) ICU VSD (217)	SSI	hypertension, PAD, previous hospitalization and surgeries	

M—male, F—female, PUS—possible unconfirmed source, BTC—bronchial tree colonization, VAP—ventilator-associated pneumonia, UTI—urinary tract infection, CRBSI—catheter-related bloodstream infection, ICU—intensive care unit, VSD—vascular surgery department, COPD—chronic obstructive pulmonary disease, CKD—chronic kidney failure, DM—diabetes mellitus, PAD—peripheral artery diseases, IO—first outbreak, IIO—second outbreak.

**Table 2:** Clinical background, antimicrobial resistance, biofilm formation, and clonality of *A. baumannii* strains examined in this study.

Strain No.	Outbreak No.	Date of Isolation	EUCAST Susceptibility Interpretation												MBL Phenotype	KPC phenotype	bla OXA Gene		MBL Gene	Biofilm Formation	Genotyping	
			Zone Diameter Breakpoint (mm) (S/R)											MIC Breakpoint (mg/L) (S/R)			OXA type	ISAba 1			PFGE	MLST
			CAZ	SXT	AK	GN	NET	TOB	CIP	LEV	IMP	MEM	TGC	CT								
PUS	unclassified	18.07.2017	6/R	6/R	6/R	6/R	6/R	6/R	6/R	6/R	6/R	6/R	6/R	2.0/S	*	*	*	*	*	*	*	*
252/17	I	26.09.2017	6/R	6/R	6/R	6/R	6/R	6/R	6/R	6/R	6/R	6/R	6/R	2.0/S	-	-	51/23/24	-	-	+	A	ST2
262/17	I	28.09.2017	6/R	6/R	6/R	6/R	6/R	6/R	6/R	6/R	6/R	6/R	6/R	1.0/S	-	-	51/23/24	-	-	+	A	ST2
279/17	I	02.10.2017	6/R	6/R	6/R	6/R	6/R	6/R	6/R	6/R	6/R	6/R	6/R	4.0/R	-	-	51/23/24	-	-	+	A	ST2
282/17	I	02.10.2017	6/R	6/R	6/R	6/R	6/R	6/R	6/R	6/R	6/R	6/R	6/R	2.0/S	-	-	51/23/24	-	-	+	A	ST2
342/17	I	16.10.2017	6/R	6/R	6/R	6/R	6/R	6/R	6/R	6/R	6/R	6/R	6/R	2.0/S	-	-	51/23/24	-	-	+	A	ST2
343/17	I	16.10.2017	6/R	6/R	6/R	6/R	6/R	6/R	6/R	6/R	6/R	6/R	6/R	2.0/S	-	-	51/23/24	-	-	+	A	ST2
279/18	II	19.03.2018	6/R	6/R	6/R	6/R	6/R	6/R	6/R	6/R	6/R	6/R	6/R	0.5/S	-	-	51/23/24	-	-	+	B	ST2
284/18	II	19.03.2018	6/R	6/R	6/R	6/R	6/R	6/R	6/R	6/R	6/R	6/R	6/R	0.5/S	-	-	51/23/24	-	-	+	B	ST2
285/18	II	23.03.2018	6/R	6/R	6/R	6/R	6/R	6/R	6/R	6/R	6/R	6/R	6/R	0.5/S	-	-	51/23/24	-	-	+	B	ST2

PUS—possible unconfirmed source, CAZ—ceftazidime, SXT—trimethoprim-sulfamethoxazole, AK—amikacin, GN—gentamicin, NET—netilmicin, TOB—tobramycin, CIP—ciprofloxacin, LEV—levofloxacin, IMP—imipenem, MEM—meropenem, TGC—tigecycline, CT—colistin, R—resistant, S—susceptible, MIC—minimum inhibitory concentrations, PFGE—pulsed-field gel electrophoresis, MLST—multi-locus sequence typing, *—not tested due to lack of bacterial strain.

## Data Availability

The data presented in this study about *A. baumanii* phenotypic and genetic characteristics are available on request from the corresponding author. Restrictions apply to the availability of data from epidemiological investigation, because the data was obtained from infection control team (ICT) and the ICT manages and stores this data.
